# Development of a dynamic optimization framework for waste management systems

**DOI:** 10.1016/j.mex.2020.101203

**Published:** 2021-01-07

**Authors:** Mohamed Abdallah, Sadeque Hamdan, Ahmad Shabib

**Affiliations:** a Department of Civil and Environmental Engineering, University of Sharjah, Sharjah, United Arab Emirates; b Laboratoire Génie Industriel, Université Paris-Saclay, CentraleSupélec, 91190 Gif-sur-Yvette, France

**Keywords:** Optimization, Mathematical modelling, Energy recovery, Financial feasibility, Carbon Footprint

## Abstract

Waste to energy (WTE) technologies have emerged as an alternative solution to municipal solid waste management. WTE systems provide major environmental and economic benefits by converting waste into accessible energy, as part of an integrated solid waste management (ISWM) strategy. However, previous studies showed that establishing an ISWM strategy based on a single type of WTE systems does not necessarily realize maximum benefits. Hence, optimizing the selection of WTE systems as part of a hybrid waste management strategy can potentially achieve maximum benefits and minimize negative impacts. However, such task is challenging due to the various alternatives and objectives, particularly those related to the material and energy recovery systems. This article presents the methods used to develop a systematic optimization framework that identifies the most beneficial set of ISWM systems through mathematical modelling. The methods include the procedures of the established framework, including base model computations, as well as the comprehensive modelling and optimization methods.

• *The energy recovery, carbon footprint, and financial profitability are computed for selected WTE facilities*.
• *The multi-objective mathematical programming is solved using the weighted comprehensive criterion method (WCCM)*.
• *The model is implemented in CPLEX software using mathematical programming language (OPL)*.

## Graphical abstract

**Figure:**
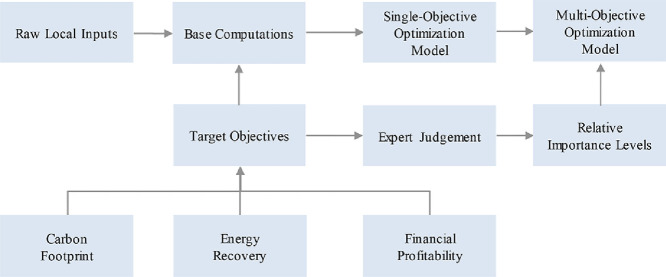

Specifications table

**Table:** 

Subject Area:	Engineering
More specific subject area:	*Solid Waste Management and Operation Research*
Method name:	*Multi-Objective Optimization Model for Integrated Waste Management Strategies*
Name and reference of original method:	*N/A*
Resource availability:	https://www.ibm.com/ae-en/analytics/cplex-optimizer

## Method details

 

## Optimization framework

**Fig. 1:**
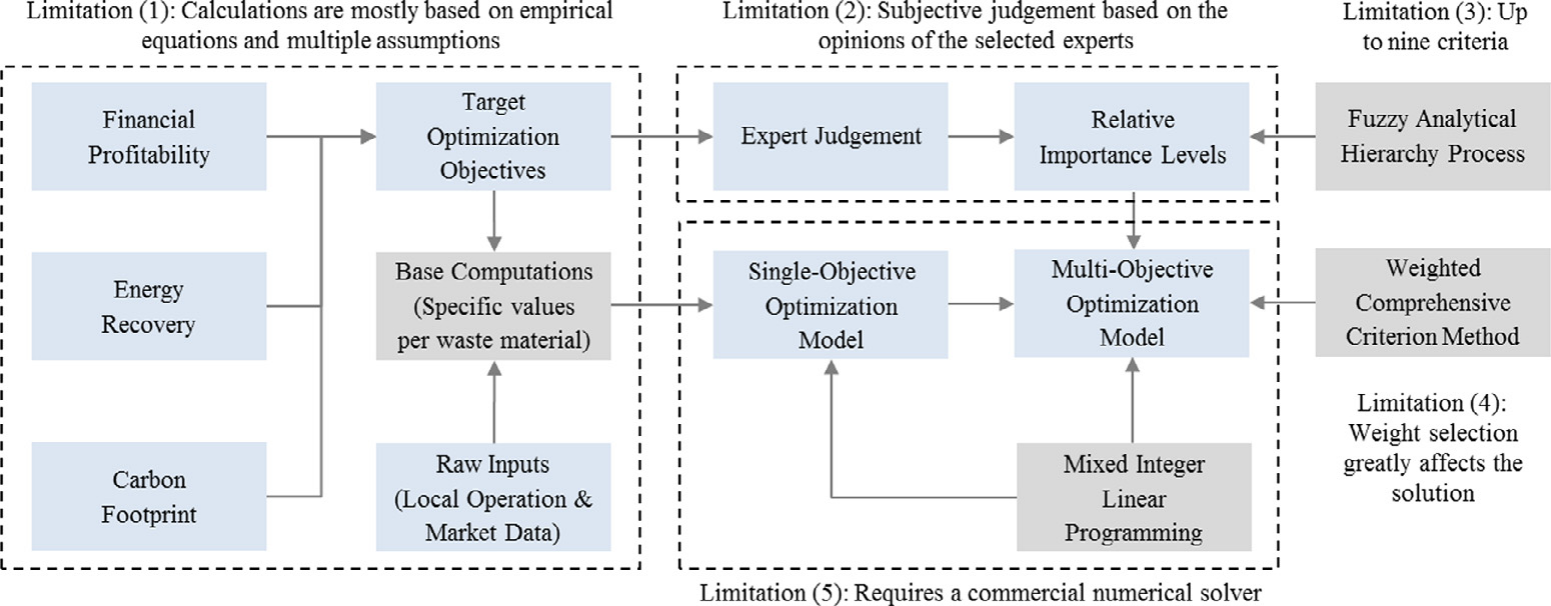
Proposed framework of the multi-objective optimization model.

This article presents the methods used to establish the framework of a multi-objective optimization model developed to systematically design an optimal waste to energy (WTE)-based management strategy for a given study area. Fig. 1 shows the optimization framework developed for the model, along with the limitations of various steps. The framework is mainly divided into two parts: model computations as well as modelling and optimization. The model computations include base calculations of energy production, carbon footprint, and financial profitability for various waste materials processed in selected waste management facilities. The modelling and optimization module includes a multi-objective mixed integer linear programming model. The multi-objective formulation is solved using the weighted comprehensive criterion method (WCCM).

## Model computations

The computations conducted on the optimization model inputs include the energy recovery, greenhouse gas (GHG) emissions, and financial profitability for each waste management facility, namely incinerator, anaerobic digester (AD), and sanitary landfill with gas recovery. The calculation steps, equations, and default values (DV) of these facilities are listed below.

### Incinerator

1- Calculate the equivalent carbon emissions, $E_{CO_{2}}$, from incineration processes [10].
  (1) $$E_{CO_{2}}=\;W_{p}\;\times \frac{44}{12}\times \sum \left(M_{i}\times \;dm_{i}\;\times \;CF_{i}\times \;FCF_{i}\;\times \;OF_{i}\right)$$

Where E_CO2_ is the total equivalent carbon emissions in a year, Gg CO_2_-eq/year

*W_p_* is the total mass of waste processed in facility, Gg/year

*M_i_* is the mass fraction of material *i* in the waste stream

*dm_i_* is the dry matter fraction of waste material *i* (DVs in Table 1)

**Table 1:** Stoichiometric parameters, energy content, and DOC values of various waste fractions.

Parameter*		Paper	Plastic	Glass	Wood	Textiles	Organics	Metal	Others
Stoichiometric parameters	n	3.6	5.0	0.0	4.1	1.0	0.0	3.7	3.4
	a	5.8	7.1	0.0	6.1	1.7	0.0	6.4	5.6
	b	2.8	1.4	0.0	2.7	0.7	0.0	1.8	2.4
	c	0.020	0.000	0.000	0.007	0.040	0.000	0.020	0.100
Dry matter fraction (dm_i_)		0.40	–	–	0.43	0.24	0.15	–	–
Dry matter carbon fraction (CF_i_)		0.46	0.75	0.00	0.50	0.50	0.38	0.00	0.03
Fossil carbon fraction (FCF_i_)		0.01	1.00	0.00	0.00	0.20	0.00	0.00	1.00
Calorific value (Btu/kg)		14,991	30,865	0	16,094	17,857	5291	661	11,464
Degradable organic carbon (DOC_i_)		0.40	–	–	0.43	0.24	0.15	–	–

⁎ [2]: from a study conducted for the US Department of Health, Education and Welfare on different waste streams, and results were orginially reported as percentage of total mass; [9]: from the Intergovernmental Panel on Climate Change (IPCC) guidelines for national greenhouse gas inventories; [8]: compiled from full-scale WTE facilities in China.

*CF_i_* is the fraction of carbon in the dry matter of waste material *i* (DVs in Table 1)

*FCF_i_* is the fraction of fossil carbon in the total carbon of waste material *i* (DVs in Table 1)

*OF_i_* is the oxidation factor (DV=1)

2- Calculate the energy produced,EP, through incineration.
  (2) $$EP=\;W_{d}\;\times \;\eta \times \sum \left(M_{i}\times \;CV_{i}\right)$$

Where *EP* is the energy production from facility, kWh

*W_d_* is the dry weight of waste processed, kg

*η* is the efficiency of energy conversion within incinerators (DV=0.30)

*M_i_* is the mass fraction of material *i* in the waste stream

*CV_i_* is the calorific value of material *i*, kWh/kg (DVs in Table 1)

3- Calculate the net present value of the incineration facility.
  (3) $$\mathrm{NPV}=\sum_{1}^{t}\left\{\left[\left(W_{p}\;\times \;\mathrm{TF}\right)+\left(\mathrm{EP}\;\times \;\mathrm{ET}\right)-\mathrm{CAPE}X_{t}-\mathrm{OPE}X_{t}\right]\;\times \;{\left(1+i\right)}^{-t}\right\}$$

Where *NPV* is the net present value, USD

*W_p_* is the total mass of waste processed in facility, Gg/year

*TF* is the tipping fee per 1000 ton of waste, USD/Gg

*EP* is the energy production from facility, kWh

*ET* is the electricity tariff, USD/kWh

*CAPEX_t_* is the capital investment costs in year *t*, USD

*OPEX_t_* is the operational and maintenance costs in year *t*, USD

*i* is the discount rate (%)

*t* is the economic life of the project (year)

### Anaerobic digester (AD)

4- Calculate the equivalent carbon emissions, $E_{CO_{2}}$, from AD plants, as per tier 2 of the Intergovernmental Panel on Climate Change (IPCC) guidelines [9].
  (4) $$E_{CO_{2}}=\;W_{p}\;\times \;EF\times \;\left(1-R\right)\;\times GWP$$

Where E_CO2_ is the total equivalent carbon emissions in a year, Gg CO_2_-eq/year

*W_p_* is the total mass of waste processed in facility, Gg/year

*EF* is the emission factor, g CH_4_/g waste (DV=0.0008)

*R* is the fraction of CH_4_ recovered (DV=0.90)

*GWP* is the global warming potential of methane (DV=28)

5- Calculate the energy produced,EP, through AD based on the general formula of waste materials *C_n_H_a_O_b_N_c_* (modified from [7,8]).
  (5) $$EP\;=\;\sum \;\left[\frac{\left(8\;\times n\right)\;-\left(2\;\times a\right)\;-\left(4\;\times b\right)\;-\left(6\;\times c\right)}{\left(12.01\;\times n\right)+\left(1.01\;\times a\right)+\left(16.00\;\times b\right)+\left(14.01\;\times c\right)}\right]\times W_{d}\times EC_{CH_{4}}\times \eta$$

Where *EP* is the energy production from facility, kWh

*n, a, b*, and *c* are the normalized mole ratio of *C, H, O,* and *N* in waste material *i* (DVs in Table 1)

*W_d_* is the dry weight of waste processed, kg

EC_CH4_ is the energy content of methane, kWh/kg (DV=14.31)

*η* is the efficiency of energy conversion within AD plants (DV=0.30)

6- Calculate the net present value of the AD plant (similar to Step 3)

### Sanitary landfill with gas recovery

7- Calculate the equivalent carbon emissions,$\;E_{CO_{2}}$, from landfill, as per tier 2 of the IPCC guidelines [3].
  (6) $$E_{CO_{2}}\;=\;W_{p}\;\times \;\sum \left(M_{i}\;\times \;\mathrm{DO}C_{i}\right)\;\times \;\mathrm{DO}C_{F}\;\times \;\mathrm{MCF}\;\times \;F\;\times \;\left(\frac{16}{12}\right)\;\;\times \;\left(1-R\right)\;\times \;\left(1-\mathrm{OX}\right)\;\times \;\mathrm{GWP}$$

Where E_CO2_ is the total equivalent carbon emissions in a year, Gg CO_2_-eq/year

*W_p_* is the total mass of waste processed in facility, Gg/year

*M_i_* is the mass fraction of material *i* in the waste stream

*DOC_i_* is the degradable organic carbon of material *i* (DVs in Table 1)

*DOC_F_* is the fraction DOC dissimilated (DV=0.77)

*MCF* is the methane correction factor (DV=0.60)

*F* is the methane fraction in landfill gas (DV=0.50)

*R* is the fraction of methane recovered (DV=0.70)

*OX* is the oxidation factor (DV=0)

*GWP* is the global warming potential of methane (DV=28)

8- Calculate the energy produced,EP, through landfill gas recovery.
  (7) $$EP\;=E_{CH_{4}}\times R\times EC_{CH_{4}}\times 10^{6}\times \eta$$

Where *EP* is the energy production from facility, kWh

E_CH4_ is the total methane emissions in a year, Gg CH_4_/year (using Eq. (6) excluding the *(1-R)* and *GWP* terms).

*R* is the fraction of methane recovered (DV=0.70)

EC_CH4_ is the energy content of methane, kWh/kg (DV=14.31)

*η* is the efficiency of energy conversion in landfill gas combustion facilities (DV=0.30)

9- Calculate the net present value of the sanitary landfill site (similar to Step 3)

## Modelling and optimization

**Fig. 2:**
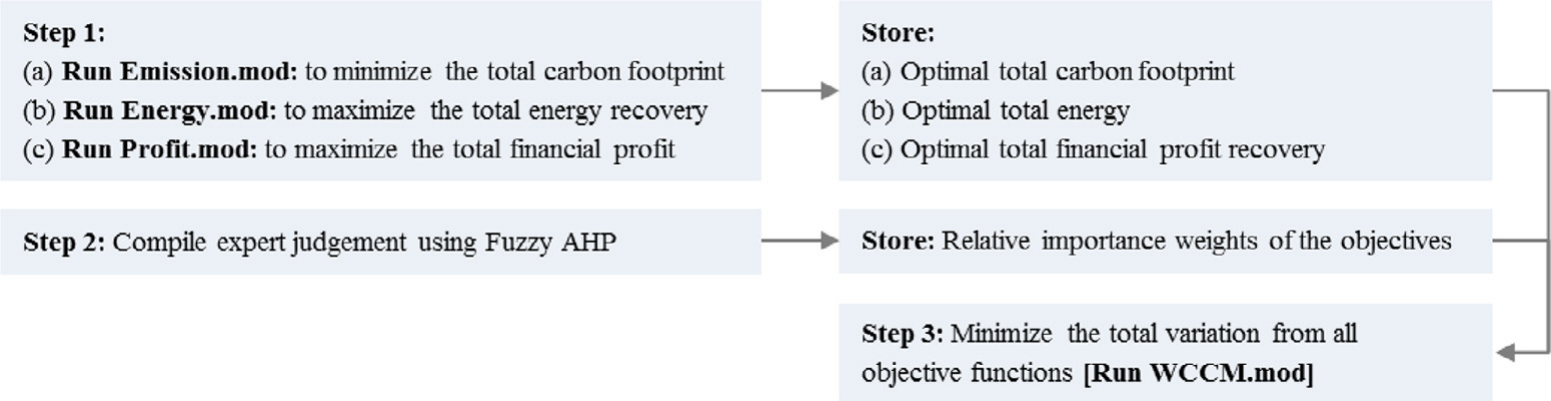
Multi-objective optimization framework.

The mixed integer linear programming model formulated in Abdallah et al. [1] is solved using the WCCM. The WCCM requires dealing with the model's objective functions individually and then developing a new objective function that combines all objectives [4], [5], [6]. Fig. 2 illustrates the process of applying WCCM for the waste management strategies. The process starts by solving the mathematical model for each objective function separately subject to all the constraints (Steps 1, 2 and 3 in Fig. 2). Next, based on the expert opinions, the importance weights of each objective function are determined using Fuzzy Analytical Hierarchy Process (AHP).

The mathematical model and the solution approach for the multi-objective formulation, the WCCM, have been implemented using the optimization programming language (OPL) in the CPLEX software (by IBM). The code files are available in the Supplementary Files. Table 2 describes the input data needed to run the model.

**Table 2:** Input data needed to run the optimization model.

Code input	Description
Demand	The quantities of waste available from each material and each year. The input data should be organized in a matrix form, where the rows are the materials (food, recyclable and non-recyclable) and the columns are the years.
CO2	The carbon footprint equivalent of each material resulting from each strategy calculated using Eqs. (1), (4), and (6). The input data should be organized in a matrix form, where the rows are the materials (food, recyclable and non-recyclable) and the columns are the strategies (anaerobic digester, incinerator, and landfill).
CO2_2	The carbon footprint equivalent of digestates and ashes resulting from each strategy calculated using Eqs. (1), (4), and (6). The input data should be organized in a matrix form, where the first row is for the digestate and the second row is for the ashes. The columns represent the strategies (anaerobic digester, incinerator, and landfill).
Energy	The energy recovery of each material resulting from each strategy calculated using Eqs. (2), (5), and (7). The input data should be organized in a matrix form, where the rows are the materials (food, recyclable and non-recyclable) and the columns are the strategies (anaerobic digester, incinerator, and landfill).
Energy_2	The energy recovery of digestates and ashes resulting from each strategy calculated using Eqs. (2), (5), and (7). The input data should be organized in a matrix form, where the first row is for the digestate and the second row is for the ashes. The columns represent the strategies (anaerobic digester, incinerator, and landfill).
CAPEX	The CAPEX value of each material under each strategy calculated using Eq. (3). The input data should be organized in a matrix form, where the rows are the materials (food, recyclable and non-recyclable) and the columns are the strategies (anaerobic digester, incinerator, and landfill).
CAPEX_2	The CAPEX value for digestates and ashes using different strategies calculated using Eq. (3). The input data should be organized in a matrix form, where the first row is for the digestate and the second row is for the ashes. The columns represent the strategies (anaerobic digester, incinerator, and landfill).
Profit_S1, Profit_S2, Profit_S3	The NPV profit for each material in each year calculated using Eq. (3). The input data should be organized in a matrix form, where the rows are the materials (food, recyclable and non-recyclable) and the columns are the years. S1, S2, S3 denote the anaerobic digester, incinerator, and landfill
Profit_Dig	The NPV profit of digestates for each strategy in each year calculated using Eq. (3). The input data should be organized in a matrix form, where the rows are the strategies and the columns are the years.
Profit_Ash	The NPV profit of ashes in each year calculated using Eq. (3). The input data should be organized in a one row, where the columns are the years.
A1, A2, A3	A single value used in the multi-objective code (WCCM.mod) representing the importance weight of the profit, carbon footprint and energy recovery objective function, respectively.
Popt	A single value used in the multi-objective code (WCCM.mod). It represents the optimal value from solving the problem for maximization of the profit objective only (Profit.mod).
Eopt	A single value used in the multi-objective code (WCCM.mod). It represents the optimal value from solving the problem for maximization of the energy recovery objective only (Energy.mod).
Copt	A single value used in the multi-objective code (WCCM.mod). It represents the optimal value from solving the problem for minimization of the carbon footprint objective only (Emission.mod).

The code files (Emission.mod, Energy.mod, and Profit.mod) should be run first in any sequence to obtain the optimal objective value for each single objective function. Then the code file (WCCM.mod) should be run to obtain the multi-objective solution.

## Conclusion

In this paper, the methodology used in the multi-objective waste management optimization problem was presented. The equations used in obtaining the input data of the mathematical model were detailed. Additionally, all software codes used to solve the formulated mathematical model were provided and thoroughly described. The codes are based on the optimization programming language of CPLEX. The presented model can be effectively utilized to generate a comprehensive waste management master plan that satisfies the specific goals of decision makers. For future research work, the analysis framework and codes can be modified to account for more features and objectives. Moreover, evolutionary methods, such as genetic algorithms, can be utilized to effectively solve the optimization problem.

## Declaration of Competing Interest

The Authors confirm that there are no conflicts of interest.

## Appendix Supplementary materials

Image, application 1

Image, application 2

Image, application 3

Image, application 4

## References

[1] Abdallah M., Hamdan S., Shabib A. (2020). A multi-objective optimization model for strategic waste management master plans. J. Clean. Prod..

[2] Arafat H.A., Jijakli K. (2013). Modeling and comparative assessment of municipal solid waste gasification for energy production. Waste Manag..

[3] Coburn J.B., Pingoud K., Thorsen G., Wagner F. (2006). Solid waste disposal. Proceedings of the IPCC Guidelines for National Greenhouse Gas Inventories Volume 5.

[4] Hamdan S., Cheaitou A. (2017). Dynamic green supplier selection and order allocation with quantity discounts and varying supplier availability. Comput. Ind. Eng..

[5] Hamdan S., Cheaitou A., Jouini O., Jemai Z., Alsyouf I., Bettayeb M. (2019). An environmental air traffic flow management model. Proceedings of the 8th International Conference on Modeling, Simulation, and Applied Optimization (ICMSAO).

[6] Marler R.T., Arora J.S. (2004). Survey of multi-objective optimization methods for engineering. Struct. Multidiscip. Optim..

[7] Ogunjuyigbe A.S.O., Ayodele T.R., Alao M.A. (2017). Electricity generation from municipal solid waste in some selected cities of Nigeria: an assessment of feasibility, potential and technologies. Renew. Sustain. Energy Rev..

[8] Ouda O.K.M., Al-Waked R., Raza S. (2014). Potential value of waste-to-energy facility in Riyadh city, Saudi Arabia. Proceedings of the 8th Jordanian International Mechanical Engineering Conference.

[9] Pipatti, R., Alves, J.W.S., Gao, Q., Cabrera, C.L., Mareckova, K., Oonk, H., Scheehle, E., Sharma, C., Smith, A., Svardal, P., Yamada, M., 2006. Chapter 4 - Biological treatment of solid. 2006 IPCC Guidel. Natl. Greenh. Gas Invent. 4.4.1-4.4.8.

[10] Rogoff M.J., Screve F. (2011). Introduction and overview. Waste-to-Energy.

